# Oligonucleotide capture sequencing of the SARS-CoV-2 genome and subgenomic fragments from COVID-19 individuals

**DOI:** 10.1371/journal.pone.0244468

**Published:** 2021-08-25

**Authors:** Harsha Doddapaneni, Sara Javornik Cregeen, Richard Sucgang, Qingchang Meng, Xiang Qin, Vasanthi Avadhanula, Hsu Chao, Vipin Menon, Erin Nicholson, David Henke, Felipe-Andres Piedra, Anubama Rajan, Zeineen Momin, Kavya Kottapalli, Kristi L. Hoffman, Fritz J. Sedlazeck, Ginger Metcalf, Pedro A. Piedra, Donna M. Muzny, Joseph F. Petrosino, Richard A. Gibbs

**Affiliations:** 1 Human Genome Sequencing Center, Baylor College of Medicine, Houston, Texas, United States of America; 2 Alkek Center for Metagenomics and Microbiome Research, Department of Molecular Virology and Microbiology, Baylor College of Medicine, Houston, Texas, United States of America; 3 Department of Molecular Virology and Microbiology, Baylor College of Medicine, Houston, Texas, United States of America; 4 Pediatrics, Baylor College of Medicine, Houston, Texas, United States of America; University of Iceland, ICELAND

## Abstract

The newly emerged and rapidly spreading SARS-CoV-2 causes coronavirus disease 2019 (COVID-19). To facilitate a deeper understanding of the viral biology we developed a capture sequencing methodology to generate SARS-CoV-2 genomic and transcriptome sequences from infected patients. We utilized an oligonucleotide probe-set representing the full-length genome to obtain both genomic and transcriptome (subgenomic open reading frames [ORFs]) sequences from 45 SARS-CoV-2 clinical samples with varying viral titers. For samples with higher viral loads (cycle threshold value under 33, based on the CDC qPCR assay) complete genomes were generated. Analysis of junction reads revealed regions of differential transcriptional activity among samples. Mixed allelic frequencies along the 20kb ORF1ab gene in one sample, suggested the presence of a defective viral RNA species subpopulation maintained in mixture with functional RNA in one sample. The associated workflow is straightforward, and hybridization-based capture offers an effective and scalable approach for sequencing SARS-CoV-2 from patient samples.

## Introduction

The COVID-19 pandemic has spread worldwide with alarming speed and has led to the worst healthcare crisis in a century. The agent of COVID-19, the novel SARS-CoV-2 coronavirus (family *Coronaviridae*), has a ~30 kb positive-sense single-stranded RNA genome. There are two large open reading frames (ORFs), ORF1a and ORF1b. ORF1a produces a large polyprotein and a joint ORF1a and ORF1b polyprotein, directly from the viral genomic RNA. Subgenomic RNAs (sgRNAs), on the other hand, are generated as a result of the discontinuous transcription from negative-stranded RNA templates and they all contain the same 5’ leader sequence [1]. Similar to other RNA viruses, coronaviruses undergo mutation and recombination [2, 3], that may be critical to understanding physiological responses and disease sequelae, prompting the need for comprehensive characterization of multiple and varied viral isolates.

To date, reports highlighting genomic variation of SARS-CoV-2 have primarily used amplicon-based sequencing approaches (e.g., ARTIC) [4–7]. Attaining uniform target coverage is difficult for amplicon-based methods and is exacerbated by issues of poor sample quality [8]. Genome variation in the amplicon primer region may also impact sequence assembly. Transcriptome characterization can further contribute to our knowledge of mutation within the SARS-CoV-2 genome, and direct RNA long read sequencing, alone and in combination with short read sequencing, have been described [1, 9, 10]. Unfortunately, these analyses are equally hampered by sample quality limitations and necessitate use of cultured cell lines.

Oligonucleotide capture (‘capture’) mitigates these issues as hybridization to specific probes not only enriches for target sequences but enables the analysis of degraded source material [11–14]. Capture enrichment has also been applied to viral sequencing, where a panvirome probe design resulted in up to 10,000-fold enrichment of the target sequence and flanking regions [15–17]. Capture based enrichment methods have been recently discussed for SARS-CoV-2 genome sequencing [18–20]. Direct RNA enrichment method has also been reported for viral genome sequencing, but each sample was enriched separately followed by pooling for sequencing [21].

Hybridization-based enrichment of RNAs can also aid in the identification of gene fusions or splice variants [13, 22, 23], which are particularly important for coronavirus biology. In addition to encoding a polyprotein that undergoes autocatalyzed hydrolysis, coronaviruses employ subgenomic RNA fragments generated by discontinuous transcription to translate proteins required for viral replication and encapsidation. These subgenomic RNA fragments share a common 62-bp leader sequence derived from the 5’ end of the viral genome, detectable as a fused junction to interior ORFs [1, 10]. Direct RNA sequencing of cultured cell lines infected with SARS-CoV-2 revealed that the junctional sequences are not evenly distributed between the ORFs [1]. How virus translation profiles from infected human patients differ from those from cultured cells is as yet unknown.

**Fig 1:**
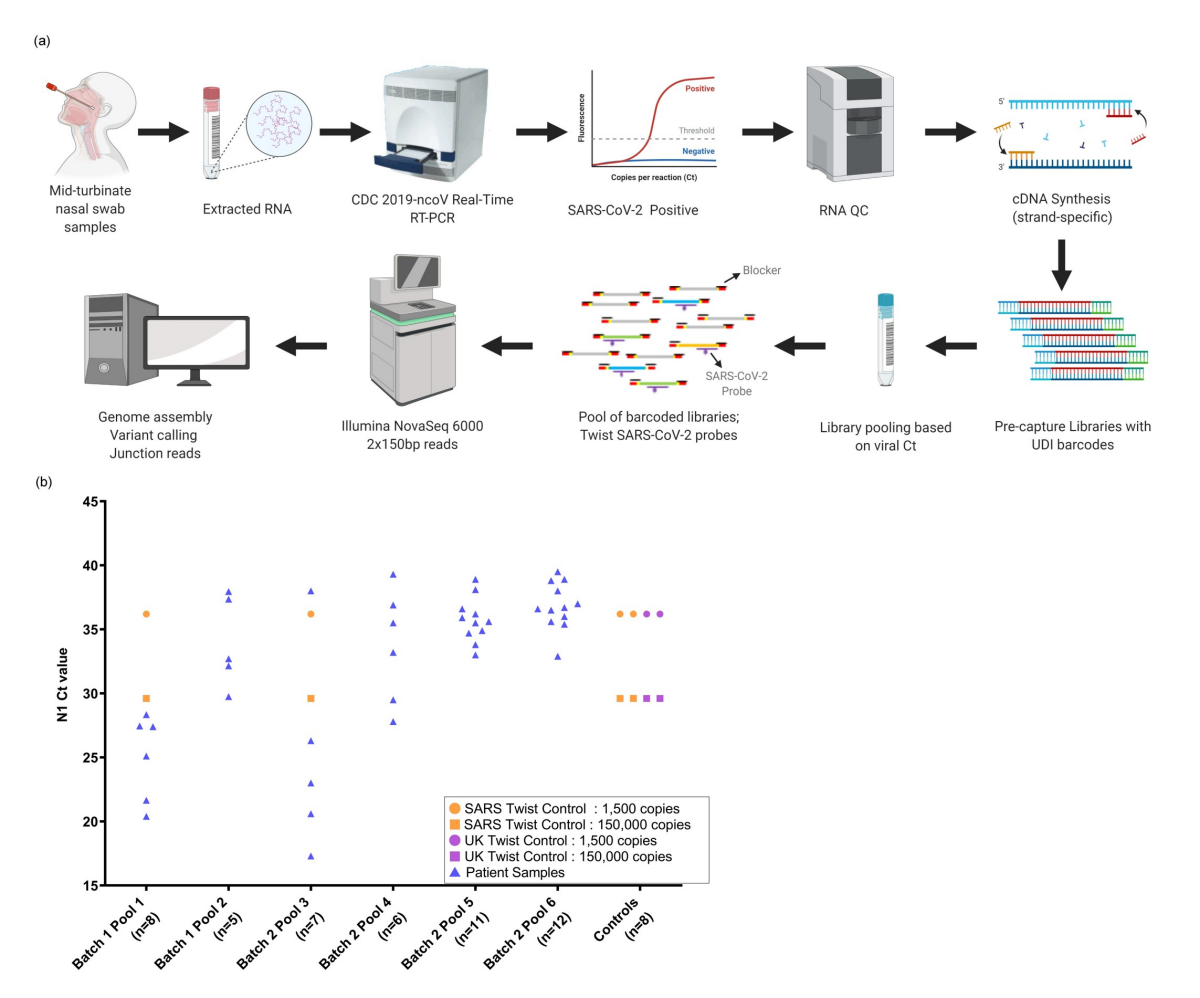
a) Schematic workflow b) Capture pools. Presented in the workflow are the different steps involved in the SARS-CoV-2 capture and sequencing methodology. Fig 1A (first row)—RNA is isolated from mid-turbinate nasal swab samples followed by Real-Time RT-PCR to detect SARS-CoV-2. Positive samples are quantified, and RNA is converted to cDNA. Fig 1A (second row)–The cDNA is used to generate Illumina libraries with molecular barcodes and these libraries are pooled based on the Ct. values into 6 pools and enriched using the SARS-CoV-2 capture probes. Additionally, Wuhan and UK strain (B.1.1.7) SARS-CoV-2 synthetic controls were hybridized as a pool or with patient samples as shown in Fig 1B. Calculated Ct values of the controls were also included. Enriched libraries were then sequenced on the Illumina NovaSeq 6000 instrument to generate 2x150 bp length reads. Data was analyzed to reconstruct genomes, identify variants and junction reads.

Here we have utilized capture probes and a streamlined workflow for sequencing and analysis of both the SARS-CoV-2 genomic sequences and of the junction reads contained within the genomic subfragments generated by discontinuous transcription (Fig 1). The method can be applied at scale to analyze clinical samples. Enriching for genomic and transcriptional RNA, followed by deep short-read sequencing, sheds light on variation in clinical SARS-CoV-2 genomic sequences and expression profiles.

## Material and methods

### COVID-19 viral testing, collection, RNA extraction and real-time reverse transcription polymerase chain reaction (RT- PCR)

The CLIA Certified Respiratory Virus Diagnostic Laboratory (ID#: 45D0919666) at Baylor College of Medicine performed real time reverse transcriptase polymerase chain reaction (RT-PCR) tests for SARS-CoV-2 on mid-turbinate nasal swab samples collected from adults presenting to the hospitals or clinics at the Texas Medical Center from March 18 through April 25, 2020. RT-PCR testing was performed as a service to Baylor College of Medicine (BCM) and affiliated institutions, while whole genome sequencing and collection of metadata was performed under an Institutional Review Board approved protocol with waiver of consent.

Viral RNA was extracted from nasal swab samples using the Qiagen Viral RNA Mini Kit (QIAGEN Sciences, Maryland, USA) with an automated extraction platform QIAcube (QIAGEN, Hilden, Germany) according to the manufacturer instructions. Starting with 140 μl of the collected sample, nucleic acids were extracted and eluted to 100 μl of elution buffer (10mM Tris-Cl, pH 8). All samples were tested by CDC 2019-Novel coronavirus (2019-nCoV) Real-Time RT-PCR Diagnostic panel [24]. Primers and probes designated as N1 and N2 targeting the SARS-CoV-2 nucleocapsid gene N were used. Samples were also tested for Ribonuclease P (RNase P) gene, to determine the quality of sample obtained. PCR reaction was set up using TaqPath™ 1-Step RT-qPCR Master Mix, CG (Applied Biosystems, CA) and run on 7500 Fast Dx Real-Time PCR Instrument with SDS 1.4 software. Samples with cycle threshold (Ct) values below 40 for both SARS-CoV-2 N1 and N2 primers were necessary to determine positivity. For seven samples with very low viral loads (N = 7); Ct >37 and <40, the RNA was concentrated 4-fold by doubling the extraction volume—280 μl and halving the elution volume—(50 μl) and submitted for sequencing.

### Sequenced samples

Forty-five mid-turbinate nasal swab samples were collected from 32 unique individuals (S1 Table). The RNA Integrity Number (RIN) values ranged from 2.3 and 5.2 with Ct values from 16–39. The amount of RNA used as input for cDNA varied from 13.6 ng to 120 ng (S1 Table). As positive controls, 1,500 (Ct = 36.2) and 150,000 copies (Ct = 29.6) of the Synthetic SARS-CoV-2 RNA from Twist Biosciences (Cat# 102024) were spiked into two 50 ng Universal Human Reference (UHR, Thermo Fisher Scientific, Cat#QS0639) RNA samples. To generate the synthetic RNA, six non-overlapping 5 kb fragments of the SARS-CoV-2 reference genome (MN908947.3) sequences were synthesized by Twist Inc. as double stranded DNA and transcribed in vitro into RNA. Three SARS-CoV-2 free mid-turbinate nasal swab samples which were negative for SARS-CoV-2 by real-time RT-PCR, were sequenced as negative controls. Due to limited sample size in this study, no other patient metadata was used to interpret results.

### cDNA preparation

cDNA was generated utilizing NEBNext^®^ RNA First Strand Synthesis Module (E7525L; New England Biolabs Inc.) and NEBNext^®^ Ultra™ II Directional RNA Second Strand Synthesis Module (E7550L; New England Biolabs Inc.). Total RNA in a 15 μl mixture containing random primers and 2X 1^st^ strand cDNA synthesis buffer were incubated at 94°C for 10 min to fragment the RNA to 200-600bp. RNA were converted to cDNA by adding a 5 μl enzyme mix containing 500ng Actinomycin D (A7592, Thermo Fisher Scientific), 0.5 μl RNase inhibitor, and 1 μl of Protoscript II reverse transcriptase, then incubated at 25°C for 10 minutes, 42°C for 50 minutes, 70°C 15 minutes, before being cooled to 4°C on a thermocycler. Second strand cDNA were synthesized by adding a 60 μl of mix containing 48 μl H_2_O, 8 μl of 10X reaction buffer, and 4 μl of 2^nd^ strand synthesis enzyme, and incubated at 16°C for 1 hour on a thermocycler. The double strand (ds) cDNA were purified with 1.8X volume of AMPure XP beads (A63882, Beckman) and eluted into 42 μl 10 mM Tris buffer (Cat#A33566, Thermo Fisher Scientific). Because these libraries were prepared primarily for sequence capture, rRNA depletion or Ploy A+ RNA isolation steps were not performed.

### Library preparation

The double-stranded cDNA was blunt-ended using NEBNext^®^ End Repair Module (E6050L, NEB). Five microliter 10X End Repair (ER) reaction buffer and 5 μl ER enzyme were added to the ds cDNA. The ER reaction was incubated for 30 minutes at 20°C on a thermocycler. After the ER reaction, cDNA were purified with 1.8X volume AMPure XP beads and eluted into 42 μl nuclease free water (129114, Qiagen). Next, 5 μl of 10X AT buffer and 3 μl of Klenow enzyme from NEBNext^®^ dA-Tailing (AT) Module (E6053L, NEB) was added to the sample. The AT reaction was incubated at 37°C for 30 minutes. After incubation, samples were purified with 1.8X volume AMPure XP beads and eluted into 33 μl nuclease free water (129114, Qiagen). Illumina unique dual barcodes adapters (Cat# 20022370) were ligated onto samples by adding 2 μl of 5uM adapter, 10 μl 5X ligation buffer and 5 μl of Expresslink Ligase (A13726101, Thermo Fisher), and incubated at 20°C for 15 minutes. After adapter ligation, libraries were purified twice with 1.4X AMPure XP beads and eluted into 20 μl H_2_O. Libraries were amplified in 50 μl reactions containing 150 pmol of P1.1 (5’-AATGATACGGCGACCACCGAGA) and P3 (5’-CAAGCAGAAGACGGCATACGAGA) primer and Kapa HiFi HotStart Library Amplification kit (Cat# kk2612, Roche Sequencing and Life Science). The amplification was carried out at 95°C for 45 seconds, followed by 15 cycles of 95°C for 15 sec, 60°C 30 seconds, and 72°C 1 minute, and 1 cycle at 72°C for 5 minutes. The amplified libraries were purified with 1.4X AMPure XP beads and eluted into 50 μl H_2_O. Quality assessment of the libraries were done using a Fragment Analyzer, DNA7500 kit (5067–1506, Agilent Technologies). The library yields were determined based on 200-800-bp range.

### Capture enrichment and sequencing

cDNA libraries with Illumina adaptors constructed from SARS-CoV-2 positive individuals were pooled into six groups (S1 Table, Fig 1B). Pools 1 and 2 were from batch 1 and pools 3–6 were from batch 2. The RT-qPCR Ct value of virus N gene varied in these pools as follows: Pool 1 with 6 samples (Ct 20.4–28.34); Pool 2 with 5 samples (Ct 29.75–37.95; Pool 3 with 5 samples (Ct 17.3–38); Pool 4 with 6 samples (Ct 27.8–39.3); Pool 5 with 11 samples (Ct 33–38.9) and Pool 6 with 12 samples (Ct 32.9–39.5). Pooled cDNA pre-capture libraries were hybridized with biotin-labelled probes from the SARS-CoV-2 Panel (Twist Biosciences, Inc) at 70°C for 16 hours according to “Protocol NGS Custom Panel Hybridization Rev1_01Feb19”. Total probe length was 120 kb and has approximately 1,000 probes and designed based on SARS-CoV-2 genome (GenBank: MN908947.3, Wuhan-Hu-1 strain). Captured virus targets were incubated with streptavidin beads for 30 minutes at room temperature. Streptavidin beads bound with virus targets were washed and amplified with KAPA HiFi HotStart enzyme. The amount of each cDNA library pooled for hybridization and post-capture amplification PCR cycles (12~20) were determined empirically according to the virus titer (N1 Ct value). In general, between 1.8–4.0 μg pre-capture library was used for hybridization with the SARS-CoV-2 probes and the post capture libraries were sequenced on Illumina NovaSeq S4 flow cell, to generate 2x150 bp paired-end reads. To evaluate the effect of hybridization-based enrichment 9 samples were sequenced before and after capture enrichment.

To ensure that the capture enrichment methodology can be used successfully for sequencing of emerging SARS-CoV-2 variants, the Twist synthetic SARS-CoV-2 RNA control 14 (GenBank ID: EPI_ISL_710528; GISAID ID: England/205041766/2020 from B.1.1.7 lineage and known as 20I/501Y.V1 or SARS-CoV-2 VUI 202012/01 [25]) and Control 2 (GenBank ID: MN908947.3, Wuhan-Hu-1) were co-captured (S2 Table). Each of these RNA controls were spiked into the 50 ng of UHR that served as the background RNA. For enrichment, two different SARS-CoV-2 genomes copies (1,500 and 150,000 copies) were used and tested in duplicates, for a total of 8 samples as shown in Fig 1B.

### Data analysis

#### Sequence mapping, genome reconstruction and variant calling

Raw fastq sequences were processed using BBDuk (https://sourceforge.net/projects/bbmap/; BBMap version 38.82) to quality trim, remove Illumina adapters and filter PhiX reads. Trimming parameters were set to a k-mer length of 19 and a minimum Phred quality score of 25. Reads with a minimum average Phred quality score below 23 and length shorter than 50 bp after trimming were discarded. The trimmed fastqs were mapped to a combined PhiX (standard Illumina spike in) and human reference genome (GRCh38.p13; GCF_000001405.39) database using a two-step BBTools approach (BBMap version 38.82). Briefly, the trimmed reads were first processed through the bloomfilter script, with a strict k = 31 to remove reads identified as human. The remaining reads were mapped to the reference genome with BBMap using a k-mer length of 15, the bloom filter enabled, and fast search settings in order to determine and remove hg38/PhiX reads. Trimmed and human-filtered reads were then processed through VirMAP [26] to obtain full length reconstruction of the SARS-CoV-2 genomes. SPAdes assembler [27] was also used for genome reconstruction. The resulting assemblies were compared to those from VirMAP. A reconstructed genome with >99% the length of the SARS-CoV-2 reference genome, NC_045512.2, was considered a fully reconstructed genome. Plots were generated using R (version 3.6.1) and the tidyverse (version 1.3.0) and ggplot2 (version 3.2.1) packages. Alignments and reference mapping were done using mafft [28] (version 1.4.0) and BBMap (version 38.82). Sequence variation of assembled genomes compared to SARS-CoV-2 reference genome (NC_045512) was analyzed by generating a genome alignment with the Mafft Multiple Alignment (version 1.4.0) plugin in the Geneious software (version 2021.1.1). For heterozygous variant analysis, the sequence reads were aligned to the reference genome using BWA-mem [29] with default parameters, realigned using GATK [30], and variants were called using Atlas-SNP2 [31]. Atlas-SNP2 was run with minimum read depth of 10, maximum read depth of 1 million along with other default parameters for variant calling. Atlas-SNP2 allows both homozygous and heterozygous variants calls. More information about the variant caller can be found in the Atlas-SNP2 [32] paper.

Variant annotation was performed with SnpEff [33] Lineage assignment of SARS-CoV-2 following Rambaut et al. (2020) used the Pangolin COVID-19 Lineage Assigner (https://pangolin.cog-uk.io). Ribosomal RNA reads were removed computationally.

#### Subgenomic mRNA and junction reads analysis

Subgenomic RNAs in Illumina reads were analyzed using Periscope program ([34], downloaded on June 21, 2021) with “–technology illumina” option. From each sample, one million reads were selected using seqtk, version 1.3 (https://github.com/lh3/seqtk). The abundance of subgenomic RNA from periscope was normalized to per million mapped reads.

#### Minimum sequence data requirements

To estimate the minimum number of reads required for full-length genome reconstruction and junction read characterization from a SARS-CoV-2 sample, data from four patient samples - 192000251D (N1 Ct 16.8), 192000440D, (Ct 22.3), 192000254D (Ct 28.7) and 192000051B (Ct 32.2) were down sampled and genome coverages and presence of junction reads were analyzed. Samples with this range of Ct values (Ct < 33) have consistently generated full-length genomes from patient samples in the sequenced set. For the down sampling analysis, from each of these samples, 1, 2, 4 and 8 million reads were randomly selected from the original fastq’s.

All scripts pertaining to the analysis has been made available via GitHub: https://github.com/BCM-GCID/Publications

## Results

A total of 45 samples collected from 32 patients between March 18 and April 25, 2020 in Houston, TX, USA were analyzed. These were a subset of individuals tested for the presence of SARS-CoV-2 early during the pandemic. RNA fractions were isolated from viral transport media and converted to cDNA. SARS-CoV-2 cDNA libraries were pooled into six groups (S1 Table). All 45 capture-enriched and nine of the pre-capture libraries were sequenced on an Illumina platform based on details provided in the online methods. A schematic workflow is shown in Fig 1A.

### Sequencing results and capture enrichment efficiency

**Fig 2:**
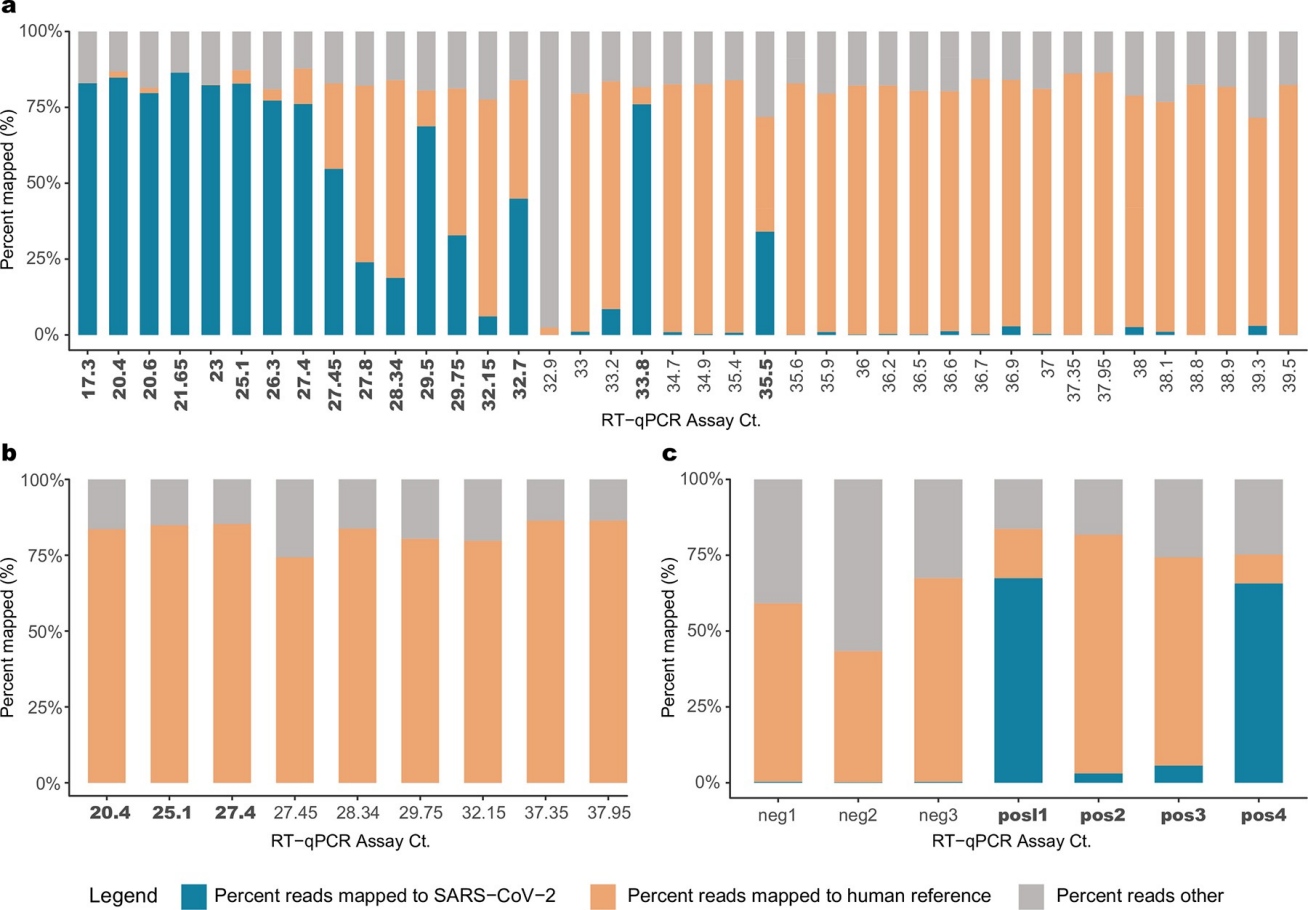
Sequence data. Ct value vs percent raw sequencing reads mapped to SARS-CoV-2 in (**a**) Capture enriched samples; (**b**) Pre-capture samples; (**c**) Positive and Negative controls. Percentage of reads mapped to the ‘SARS-CoV-2’ genome, to the ‘human’ reference genome and a third category called ‘reads others’, which is the combined total of trimmed reads and reads that do not fall under the two other categories are plotted in this figure. Ct values in bold indicate samples that provided full-length genome assemblies.

A total of 7.15 billion raw reads were generated for the 45 SARS-CoV-2 positive samples sequenced (S1 Table). Since this study was to optimize the methodology, samples were sequenced deeper to ensure that results among samples were not biased. Sequences were trimmed to filter low quality reads and subsequently mapped to the GRCh38 reference genome to identify human reads (Fig 2A). Trimmed non-human sequence reads were analyzed using the VirMAP [21] pipeline where average 23.91 percent of total reads with a standard deviation of 33.43% from post-capture libraries mapped to the SARS-CoV-2 reference. One sample (192000446B), which had only 6.37 ng total RNA starting material, did not generate any SARS-CoV-2 reads. Overall, the percentage of reads represented by SARS-CoV-2 was higher in samples with CDC protocol-based RT-qPCR Ct values <33 (Fig 2A).

To estimate the capture enrichment efficiency, pre-capture libraries for nine samples, ranging in Ct values of 20.4 to 37.95 (i.e., high to low titer in the original samples), were also sequenced, generating 152.1–322.9 million reads per sample. Samples 192000106B and 192000090B, with Ct > 37 produced zero reads mapping to the SARS-CoV-2 reference genome. In the remaining seven samples, less than 0.022% of reads were deemed SARS-CoV-2 (Fig 2B). Collectively, capture enrichment increased the SARS-CoV-2 mapping rate to 50.9%, a 9,243-fold mean enrichment.

Spiked synthetic SARS-CoV-2 RNA, encompassing six fragments of 5 kb each, served as a positive control and were enriched successfully at both 1,500 and 150k copies per sample (Fig 2C). Enrichment as indicated by higher percentage of read alignment to the SARS-CoV-2 genome was observed in both the 1,500 copy libraries (n = 2) and the 150k copy libraries (n = 2) at 3–5% and 65% respectively (S1 Table). This translates to an approximate 91,858-fold enrichment in the 1,500 copy libraries and 13,778-fold enrichment in the 150k copy libraries compared to their starting amounts in the RNA. Three SARS-CoV-2 PCR negative samples were also sequenced, where <0.5% of reads mapped to the SARS-CoV-2 reference genome at 3–5 locations that are not reproducible across samples (S1 Table; S1 Fig).

The control RNA libraries consisting of either Twist B.1.1.7 (UK) or the Wuhan control sample, generated 34.7–80.4 million reads and 30.8–60.1 million reads respectively (S2 Table).

### Genome reconstruction and genomic variations

To assess the ability of the capture methodology to assemble full-length genomes, both the nine pre-capture and 45 post capture libraries were assembled using both the VirMAP pipeline and the SPAdes *de novo* assembler [27].

**Fig 3:**
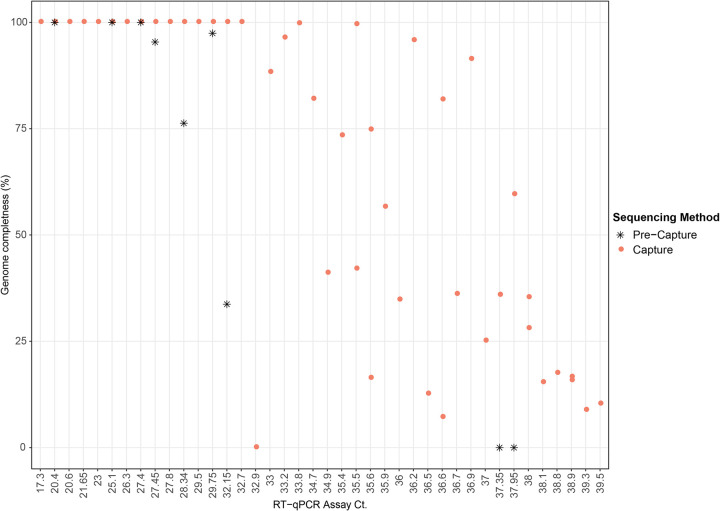
Scatter plot showing genome completeness as a function of Ct value. Pink circles represent post-capture samples and black asterisks represent pre-capture samples.

Full-length SARS-CoV-2 genomes were obtained from 17 of the 45 capture-enriched samples. Genome coverage in these 17 samples varied from 1071x to 3.19x million (S1 Table). Successful full-length genome assembly corresponded with Ct values below 33 (Fig 3), regardless of the total reads generated during sequencing. No variability between samples due to random priming of the cDNA synthesis or gaps in genome coverage were noticed using this method (S2 Fig). Two samples with Ct values above 33, 192000296 (Ct 33.9) and 192000354 (Ct 35.5), obtained from a single patient, also yielded full-length genome reconstructions with acceptable quality (N ≤ 0.5%, where N is the undetermined nucleotide). Partial genome reconstructions were achieved for the remaining samples, there was no clear trend observed between the percentage of the genome reconstructed and the Ct value when Ct values were above 33 (Fig 3). Full-length genome sizes of the 17 capture-enriched and assembled sequences varied from 29.68 kb to 30.15 kb (S3 Fig). The number of variants observed relative to the SARS-CoV-2 reference genome sequence NC_045512.2, which includes single nucleotide polymorphisms and a single indel, ranged from 5 to 15 per sample, with a mean of nine per sample.

**Fig 4:**
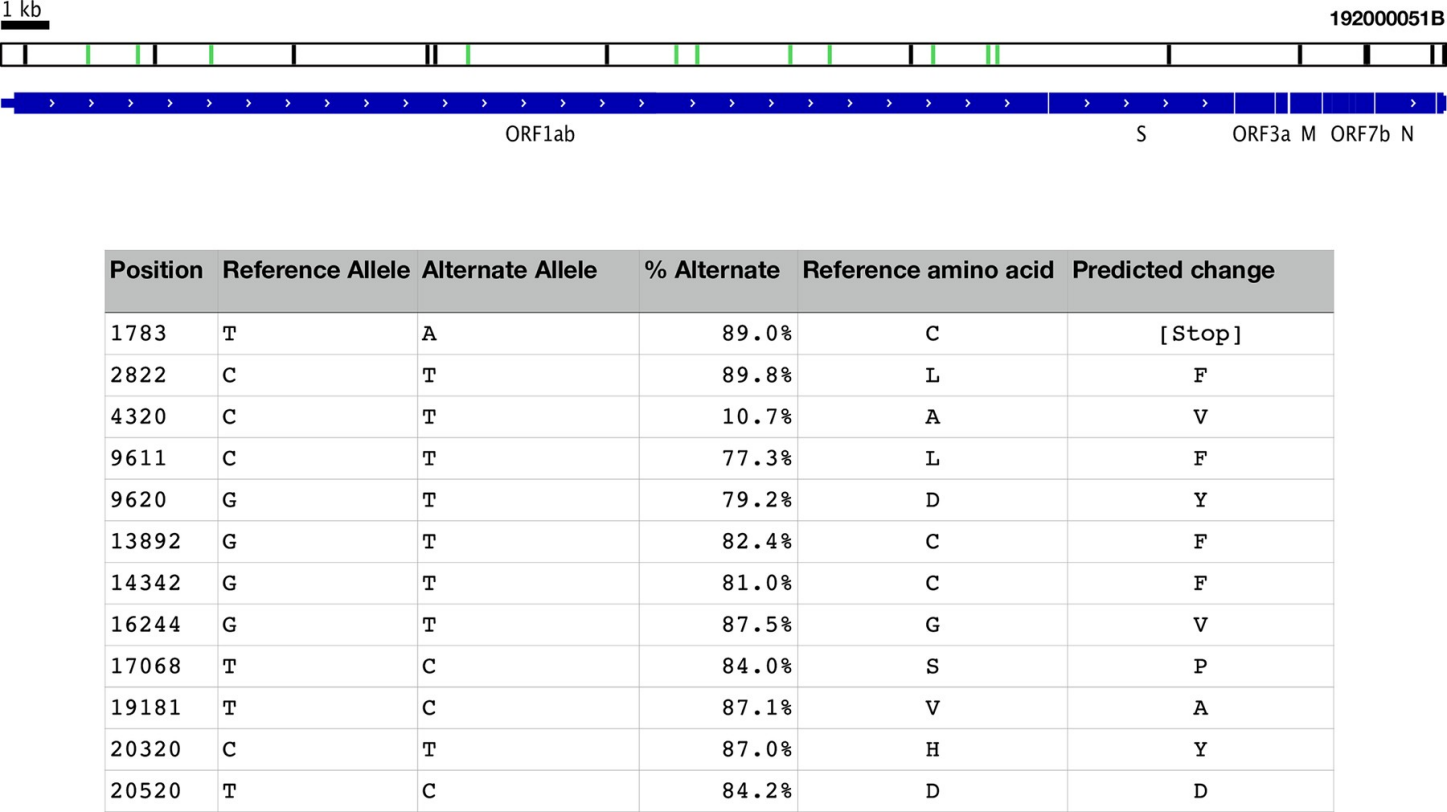
Schematic representation of 192000051B assembly. Black bars represent loci where the assembly called alleles different from the NCBI reference sequence NC_045512. Green bars represent mixed loci where both reference and alternative alleles were called. All mixed loci are in the ORF1ab gene, and are listed in the table, along with the frequency of the alternate allele at the position, and the predicted effect in translation.

Out of the nine pre-capture samples, three (192000072B, 192000021B, 1920000003B), all with Ct values < 27.4, yielded full-length genomes with 28x – 265x genome coverage and partial genome reconstructions were generated for the remaining four samples with a genome coverage of 1 – 6x. SARS-CoV-2 reads were not detected in the last 2 samples. Alignment of DNA sequence reads from one sample (192000051B) to the reference SARS-CoV-2 genome sequence NC_045512.2 that is based on the first published isolate from Wuhan SARS-CoV-2 reference genome, revealed multiple alleles (Fig 4; S4 Fig). Three samples were identified with a fraction of reads representing the ’A’ lineage [35] (S3 Table). Further investigation of the clinical correlates of this observation are underway. This study found the prevalent A > G mutation (nt position 23403) which results in the D614G amino acid change among twenty-three of the 28 capture enriched samples [36]. To evaluate the use of capture methodology to sequence new SARS-CoV-2 variants, B.1.1.7 variant synthetic RNA were capture enriched with the Wuhan probe set in the same pool with Wuhan strain synthetic RNA and these libraries were sequenced. Analysis of the control capture libraries run in duplicate of the Twist UK control at 1,500 and 150k copies showed presence of all alleles 13 nonsynonymous, 1 nonsense, and 3 deletion mutations, characteristic of the B.1.1.7 variant with correct lineage assignment using Pangolin (S2 Table).

### Characterization of SARS-CoV-2 subgenomic mRNAs

**Fig 5:**
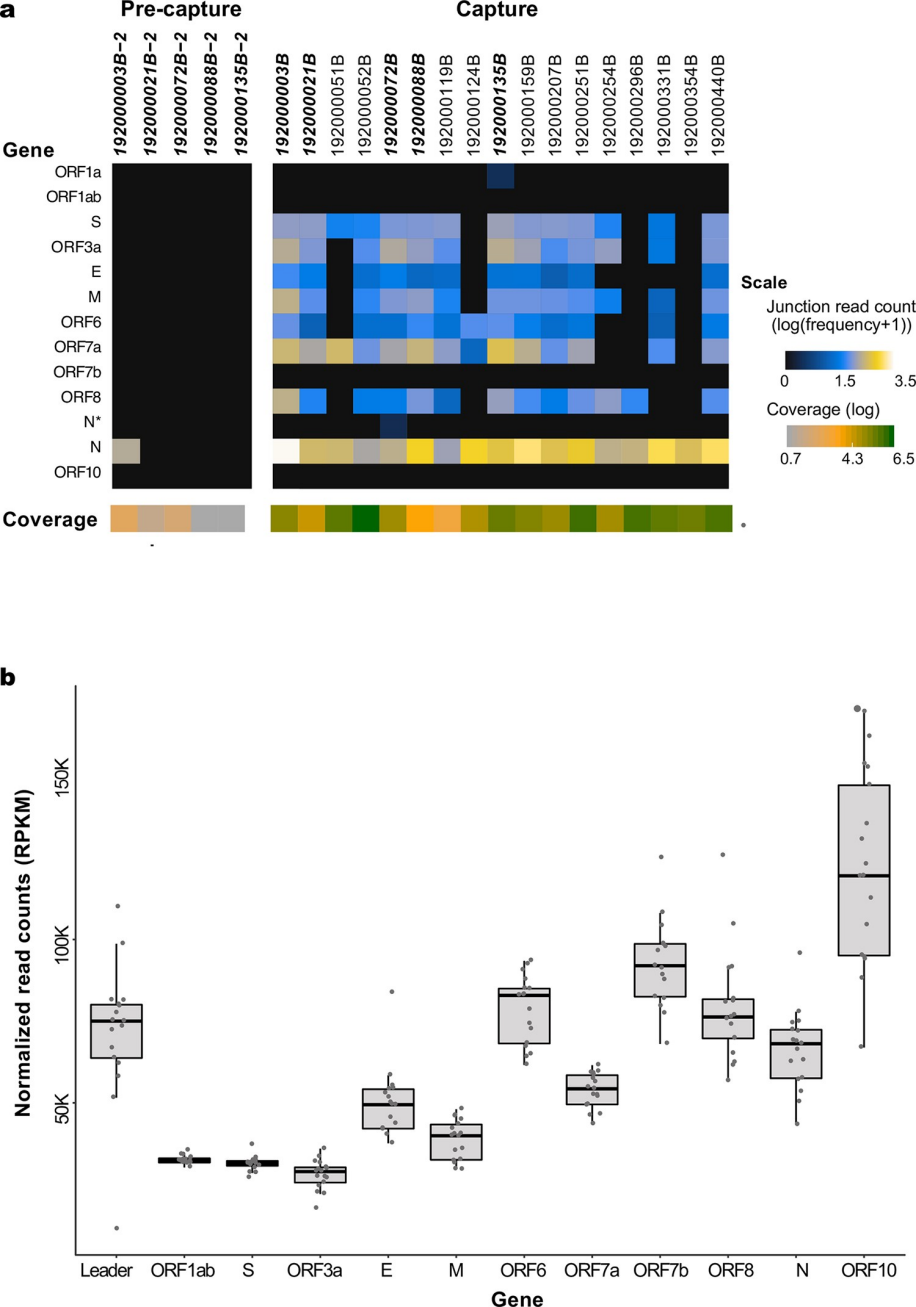
SARS-CoV-2 subgenomic mRNAs. **(a)** Junction read quantification per gene (log transformed), estimated using one million reads/sample from five pre-capture and 17 capture samples. Samples chosen for this analysis have above 95% genome completeness. The coverage level per sample is shown below the gene heatmap. Samples in bold denote same sample sequenced as pre-capture and capture. (**b**) ORF read coverage shown as normalized read counts (RPKM) per gene for 17 capture samples. The boxes represent the first quartile to the third quartile of the normalized read counts for each ORF. The horizontal lines in the boxes represent the median values, and the whiskers represent the variability outside the lower and upper quantiles.

To identify and quantitate subgenomic mRNAs, reads were aligned to the SARS-CoV-2 reference genome NC_045512.2. Only samples with full-length genomes (N = 17 capture and N = 5 pre-capture) were analyzed for junction reads to avoid introduction of any bias in identifying subgenomic RNA due to gaps in sequence coverage (Fig 5A and S4 Table). While full-length genomes were reconstructed from three pre-capture samples, an additional two samples with >95% genomes reconstructed, 192000135B (with 97.4%) and 192000088B (95.3%), were also included in this comparison (Fig 5A and in S4 and S5 Tables). To characterize ORF expression in the capture and pre-capture libraries, one million reads/sample were calculated and plotted in Fig 5A (see details in S4 and S5 Tables). Among the five pre- and post-capture comparison pairs, with the exception of N gene in one sample (192000003B_2) junction reads were identified only in libraries after capture enrichment.

In the capture libraries, junction reads were identified in a million reads in all 17 samples in the N gene, followed by S gene and ORF7A in 14 samples, ORF3a, M, ORF6 and ORF8 in 13 samples, E gene in 11 samples, while in the rest of the samples, junction reads were either detected in just one sample (ORF1a) or none (ORF1ab, ORF7b and ORF10) in these samples. In one sample 192000072B, Periscope program identified a previously reported non-canonical sgRNA (N*) [34]. The average number of junction reads in million reads/sample was the highest for N gene (819.7), followed by ORF7a (201.2) ORF3a (129.3) and S gene (110.2). Log transformed values are shown in Fig 5A. For the remaining ORFs, the average was less than 100 junction reads/million. Among the 17 libraries with full-length genomes, there is only one pair 192000296B (Ct 33.8) and 192000354B (Ct 35.5), sampled twice from the same subject (Patient #12) and the junction read expression for N gene was detectable in both of these samples (S4 Table) but not ORF8. Junction reads were completely absent in the Twist synthetic RNA samples which were six non-overlapping synthetic RNA fragments of 5kb each and does not carry any subgenomic RNAs with leader sequence. This supports our observations that finding junction reads from capture reads is not due to an experimental artifact. Subgenomic RNAs were also absent in the three nasal swab samples that were negative for SARS-CoV-2.

There were no gaps in the ORF read coverage in any of the 17 capture samples (Fig 5B). From 5’ to 3’ of the genome, there was a gradual increase in the read coverage for the genomic and subgenomic (transcriptomic) RNA reads as all sgRNAs originate from the 3′ end. Across the genes in these 17 samples, ORF1ab and ORF3a had the lowest reads per kilobase million (RPKM) values (average 32509 and 27957 RPKM, respectively) while the highest values were seen for ORF10 with a count of 121,643 (Fig 5B).

### Data down sampling

Using 1 million reads per sample, among the four samples, the sample 192000051B (N1 Ct 32.2) had the lowest SARS-CoV-2 genome coverage where 99.7% of the bases were covered at a minimum of 20x coverage, which will allow detection of variants. Assemblies were successfully reconstructed for all Ct ranges using 1 million reads per sample. The lowest median genome coverage for 1 million reads per sample was in sample 192000254 (N1 Ct 28.7) at 1,312x (S6 Table). Junction reads were identified in the down sampling datasets with progressively increase in numbers from 1–8 million reads (S7 Table). The lowest detectable junction read count was 19 using 1 million reads (S7 Table) and if a junction read was not detectable in one million reads, it was not detectable in 2, 4 or 8 million reads as well. The exception to this was one instance, where in sample 192000254 in ORF7a, junction reads were not detectable with one and two million reads, but one read was found in four and three read in 8 million reads (S7 Table).

## Discussion

We employed a hybridization-based oligonucleotide capture methodology, combined with short read sequencing and data analysis for culture-free genome reconstruction and transcriptome characterization of the SARS-CoV-2 virus. The approach provided complete viral genome sequences and identified subgenomic fragments containing ORFs, shedding light on SARS-CoV-2 transcription in clinical samples. As shown with enrichment of the B.1.1.7 Twist control sample (GISAID ID: England/205041766/2020), capture enrichment can succeed in identifying variations in new strains. This method uses routine cDNA and library preparation along with Illumina sequencing, employing 96 or more barcodes. Patient samples can be pooled for capture and sequencing since the samples are barcoded during library preparation, allowing for a high-throughput method of identifying emerging viral strains.

In all samples tested, the capture method resulted in a significant enrichment of SARS-CoV-2 nucleic acids. The enrichment efficiency was optimized using two spike-in synthetic SARS-CoV-2 RNA controls against a background of human Universal Human Reference RNA (UHR, Thermo Fisher Scientific, Cat#QS0639), yielding a 91,858-fold enrichment in the 1,500 copy (Ct = 36.2) libraries and 13,778-fold enrichment in the 150k copy (Ct = 29.6) reconstructed samples. A 9,243-fold enrichment was observed for nine patient samples when the sequence data from pre and post capture libraries were compared. When pooling samples with a variety of Ct values, some unevenness in SARS-CoV-2 sequence representation was initially observed. This was resolved by pooling groups of samples based on their range of Ct values prior to capture enrichment.

Full length SARS-CoV-2 genomes were able to be assembled from 17 of the 45 samples analyzed. High quality, full-length reconstructions from capture enrichment appears to be reliably achieved with a viral Ct <33. Between a Ct of 33 and 36, the full-length genome is recovered in some samples while partial genomes, consisting of >50% of the genome length, were reconstructed for the majority (Fig 3). For SARS-CoV-2 genome sequencing, multiplex amplicon sequencing has been used the most to date which includes the primer pools designed by ARTIC consortium (V1 V2 and latest is V3) as well as a third version NIID-1 (Quick J) [4, 37]. ARTIC V1 primer set, was successful in recovery of full-length genomes only from samples with relatively high viral load (Ct < 25) in clinical qPCR tests, as certain primer pairs were determined to be under performing. The updated ARTIC consortium primer set V3 and the NIID-1 primer set redesigned the problematic primer sets and were shown to work well with Ct values in clinical qPCR from 25 to 30 [4]. A multiplex amplicon-based approach by CDC was effective in generating full length genome sequences <Ct of 33 although Ct values between 30 and 33, genome recovery varied between samples [38]. In another report, ARTIC primers were used initially for amplification of SARS-CoV-2 clinical samples and the full-length genome recovery from sequencing these amplicons were compared by different library preparation methods for Illumina sequencing [39]. They reported that samples below Ct <27 produced near full-length genomes, although from samples with Ct <30, longer and higher quality genomes were reported.

Two recent studies used capture probe sets to enrich SARS-CoV-2 from 3 [19] and 8 [18] patient samples respectively. The paper describes assembly of the SARS-CoV-2 genome by using DNA nanoballs libraries sequenced on the MGISEQ-2000 (MGI Shenzhen, China) platform. Xiao et al., in their paper, also compared the performance of the capture method with the metatranscriptome sequencing as well as the amplicon sequencing and reported better accuracy for identifying SNVs for challenging samples (Ct >29) [18]. Another published study compared three methods, NEB+Twist and Illumina capture enrichment methods and Paragon amplicon method for sequencing of the SARS-CoV-2 samples [20]. In total, 13 samples ranging in Ct values 11.3–22.6 were sequenced using all three methods. They reported better performance (greater uniformity in coverage and lower false positive rates) from capture enrichment compared to the amplicon sequencing. None of these papers have looked at the viral sub-genomic reads. The aim of this paper is to present data demonstrating the use of capture enrichment to obtain full-length genome and sub-genomic viral reads from patient samples, without the need for a culture stage, allowing to measure transcription.

Using the capture enrichment methodology, full-length genomes were obtained consistently from clinical samples up to Ct 33 from our sample set. Further, as shown from the data (S1 Table), generating more sequence data for low titer samples does not lead to full-length genome recovery. There is supporting information now based on the success rate of the culture of the SARS-CoV-2 at different Ct Values, where the probability of culturing virus declines to 8% in samples with Ct > 35 and to 6%, 10 days after symptom onset [40]. When these findings are combined with sequencing data from PCR and capture studies, it becomes clear that culturing and extracting full-length genomes from low-titer SARS-CoV-2 samples have limitations.

In sample 192000051B, capture enrichment led to the discovery of a mixed population of SARS-CoV-2 virus, which may include a putative defective viral RNA species incapable of translating the viral polyprotein encoded in ORF1ab, but which coexists with replication competent variants. ORF1ab consists of multiple loci spanning 21 kb that together encode the polyprotein essential to the replication of the viral genome contained mixed alleles. Only one of these loci (T20520C) is expected to produce a synonymous change in the coding sequence. All the other loci are predicted to change the amino acid sequence of the polyprotein. Most notable is T1783A, which introduced a stop codon early in the translation of ORF1ab. Introduced stop codons are rare among the submitted genome assemblies tracking the evolution of SARS-CoV-2 (nextstrain.org) but are distributed all along the genome (S4 Fig). In some regions, these introduced stop codon alleles occur in multiple loci along multiple lineages, one of which at a significant enough frequency to be scored with high homoplasy [41]. The low phylogenetic signal disqualifies these loci from much further analysis.

A stop codon early in the ORF1ab gene should prevent propagation of the viral genome, but it can possibly be maintained by functional copies of co-infecting replication competent virus as a mixed population. Defective viral RNA that is replicated and packaged maintained in mixed populations have been detected in other coronaviruses [42] and in dengue virus [43]. If the requirement for translational fidelity of the ORF1ab gene were lost, it would remove any selective pressure on the remainder of the gene, and may explain the accumulation of additional mutations observed in the defective species. It would not interfere with the generation of subgenomic segments of the rest of the genome for translation of the proteins necessary to package the virus. Engineered defective viruses that interfere with the replication of functional viruses are a potential antiviral tool in the treatment of respiratory virus diseases [44].

With the emergence and rapid spread of these SARS-CoV-2 variants, sequencing data for SARS-CoV-2 samples will be important for surveillance and disease spread. Capture enrichment methodology was successful in sequencing the Twist synthetic SARS-CoV-2 RNA control sample that represents that B.1.1.7 lineage using the Wuhan capture probe set. Capture enrichment was able to enrich not only the key point mutations, N501Y, A570D and P681H but also the four deletions (HV69-70del, Y144del, SGF3675-3677del and D3L) using the Wuhan capture probe set suggesting that a single probe set will be sufficient to sequence multiple SARS-CoV-2 variants.

Our capture approach enabled simultaneous detection and quantitation of the subgenomic fragments. RPKM values plotted in Fig 5B were for reads originating from both genomes and sub-genomes. Plotting of this data shows that capture is not biased in enrichment and that the increase in coverage of the reads from 5’-3’ is in agreement with the transcription pattern of the sub-genomes as described by Kim et al. [1]. Further, no junction reads were found in the Twist synthetic RNA samples which does not have segmental RNAs with junction reads. This datasets provides the supportive evidence for absence of template switching during the reverse transcription or polymerase reaction.

Kim et al. [1], reported SARS-CoV-2 quantitative expression in SARS-CoV-2 infected Vero cells (ATCC, CCL-81) based on junction reads obtained from Nanopore based direct RNA sequencing. In their study, the N gene mRNA was the most abundantly expressed, while 7b gene expression was the least, which matches to our observations in these 17 samples. For the other ORFs, which they reported S, 7a, 3a, 8, M, E, 6 as the next most abundantly expressed ORFs, in our 17 samples, it was ORFs 7a, 3a, S, M, 6 and E. There is another recent report in which sgRNAs were quantified from patient samples sequenced on the Oxford Nanopore Technologies (ONT) ARTIC data, N and M genes were the most expressed ORFs [34]. But they reported, ORF8 and ORF3a as least expressed, which is different from our study as well as Kim et al. [1]. In all three studies, N gene was reported as most expressed, which agrees with the mass spectrometry studies as the most highly expressed gene [45]. Among other ORFs, Kim et al. [1], found no subgenomic fragments enabling translation of ORF10 and it was not identified in our samples as well. In this study, we looked for junctions reads in our data and used them to quantitate ORF expression patterns in the 17 samples with full length genome reconstructions (Fig 5 and S4 and S5 Tables). We note however that the capture methodology is limited in its ability to identify the RNA modifications that were reported by Kim et al. [1].

Down sampling of the sequence data from a range of Ct values showed that one million reads per sample and up to Ct 33 was sufficient to generate a minimum of 20x genome coverage of 99.7% or greater (S6 Table). Using 1 million reads per sample, the minimum median coverage obtained was at 1,132x and assemblies were also successfully reconstructed for all Ct ranges. In the down sampling experiment, junction reads were identified with progressively increase in numbers using 1–8 million reads (S7 Table). One million reads/sample was sufficient to identify junction reads in most of the ORFs that are expressed (S7 Table).

This article was posted on bioRxiv on July 27^th^, 2020. As a follow up to this study, an additional 95 patient samples with SARS-CoV-2 Ct values of 9.3–31.3 Ct were sequenced. For all 95 samples, SARS-CoV-2, full-length genomes were reconstructed (unpublished data). This method has a straightforward work-flow and is scalable for sequencing large numbers of patient samples.

In summary, this capture enrichment and sequencing method provides an effective approach to generate SARS-CoV-2 genome and transcriptome data directly from clinical samples.

### Accession numbers

All the 17 full-length reconstructed SARS-CoV-2 genomes are available at GISAID *(**www*.*gisaid*.*org**)* under the accession numbers EPI_ISL_444022, EPI_ISL_445078—EPI_ISL_445084, EPI_ISL_501168 –EPI_ISL_501174 and EPI_ISL_513294.

## Supporting information

S1 Fig Genome coverage plots for the three SARS-CoV-2 negative samples. Coverage is localized despite the 45–91 M reads that these samples obtained post-capture.
(TIF)

S2 Fig Genome coverage plots. Genome coordinates on X-axis and coverage in log scale of Y-axis for the 17 samples with full length SARS-CoV-2 genome reconstructions.
(TIF)

S3 Fig A multiple sequence alignment (using MAFFT) of 17 reconstructed SARS-CoV-2 genomes and Wuhan-Hu-1 reference genome (NC_045512). Grey indicates agreement with the reference, black is a disagreement, and pink marks areas in the reconstruction with an ambiguous nucleotide, “N”. The pangolin lineage assignment is listed next to the sample name. The extra length of the 192000251B seen here is an assembly artifact and was excluded from analysis.
(TIF)

S4 Fig Stop codon variants in sampled SARS-CoV-2 genomic assemblies. A snapshot of full length SARS-CoV-2 genome assemblies from GISAID and NCBI on 27 May 2020 was downloaded (comprising 39246 entries), and processed to detect single nucleotide variant alleles that introduced a stop codon. Introduced stop codons were detected in 270 entries, and the frequency of these alleles are plotted along the SARS-CoV-2 reference genome position. Introduced stop codons are rare but are distributed throughout the genomic sequences. Multiple loci harbor stop codons in unrelated assemblies.
(TIF)

S1 Table Sample information, capture pools and sequencing metrics details. (XLSX)

S2 Table Capture sequencing metrics for UK B.1.1.7 control sample. (XLSX)

S3 Table Lineage analysis of the 17 full-length genomes. Lineages assigned by the tool Pangolin (https://pangolin.cog-uk.io), which follows the methods in Rambaut et al., 2020.
(XLSX)

S4 Table Junction read counts normalized to per million mapped reads from one million reads/sample identified in the post capture data of 17 samples with full-length genomes. (XLSX)

S5 Table Junction read counts normalized to per million mapped reads from one million reads/sample identified in the nine samples sequenced before (IDxxxxB-2) and after capture (IDxxxxB) enrichment. (XLSX)

S6 Table SARS-COV-2 genome coverages from 1–8 million (M) down sampled reads. (XLSX)

S7 Table SARS-COV-2 junction read counts from 1–8 million (M) down sampled reads. (XLSX)

S8 Table GISAID hcov-19 acknowledgement table. (PDF)
