# Supplementary figures and images for: Oligonucleotide capture sequencing of the SARS-CoV-2 genome and subgenomic fragments from COVID-19 individuals
Source: PLoS One. 2021 Aug 25;16(8):e0244468. doi: 10.1371/journal.pone.0244468 (PMC8386831; doi:10.1371/journal.pone.0244468)

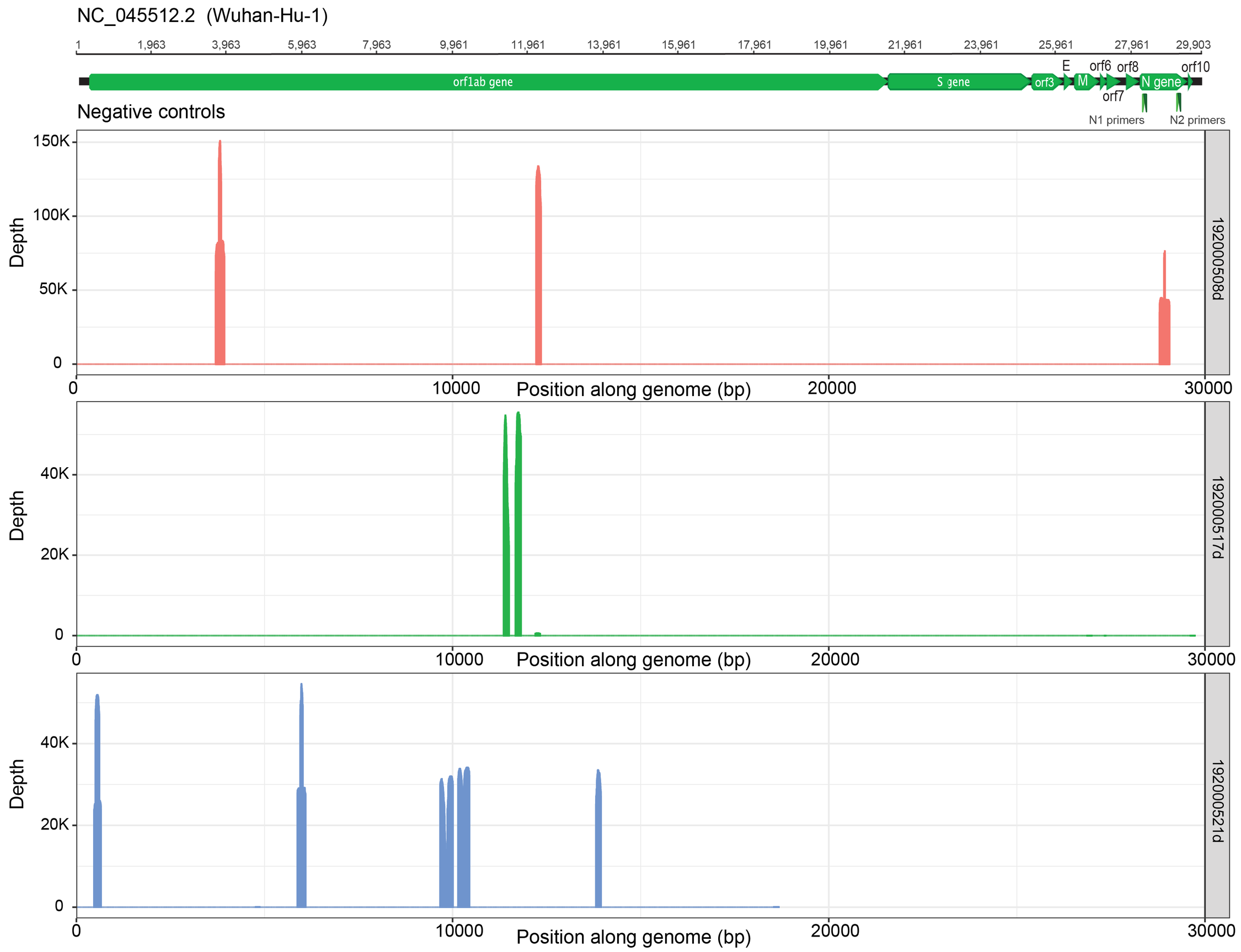

Supplement: S1 Fig — Coverage is localized despite the 45–91 M reads that these samples obtained post-capture. (TIF) [file pone.0244468.s001.tif]

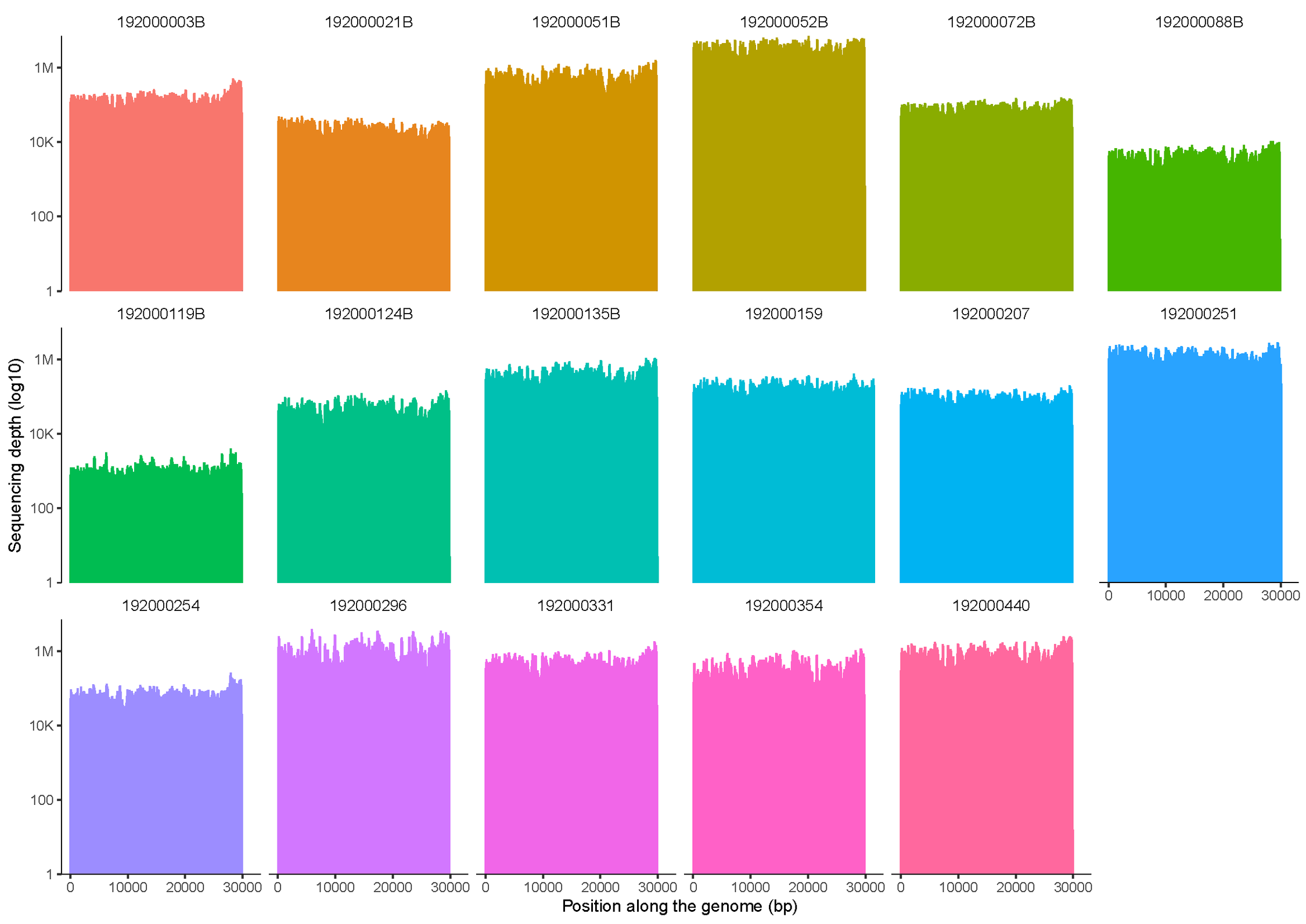

Supplement: S2 Fig — Genome coordinates on X-axis and coverage in log scale of Y-axis for the 17 samples with full length SARS-CoV-2 genome reconstructions. (TIF) [file pone.0244468.s002.tif]

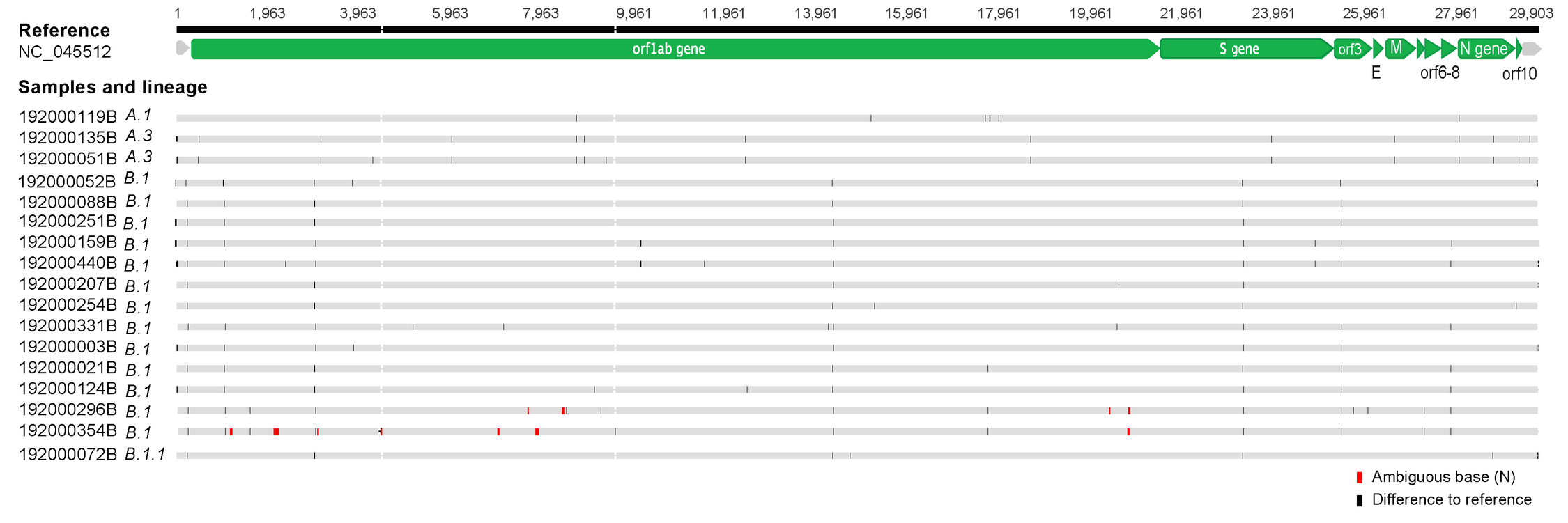

Supplement: S3 Fig — Grey indicates agreement with the reference, black is a disagreement, and pink marks areas in the reconstruction with an ambiguous nucleotide, “N”. The pangolin lineage assignment is listed next to the sample name. The extra length of the 192000251B seen here is an assembly artifact and was excluded from analysis. (TIF) [file pone.0244468.s003.tif]

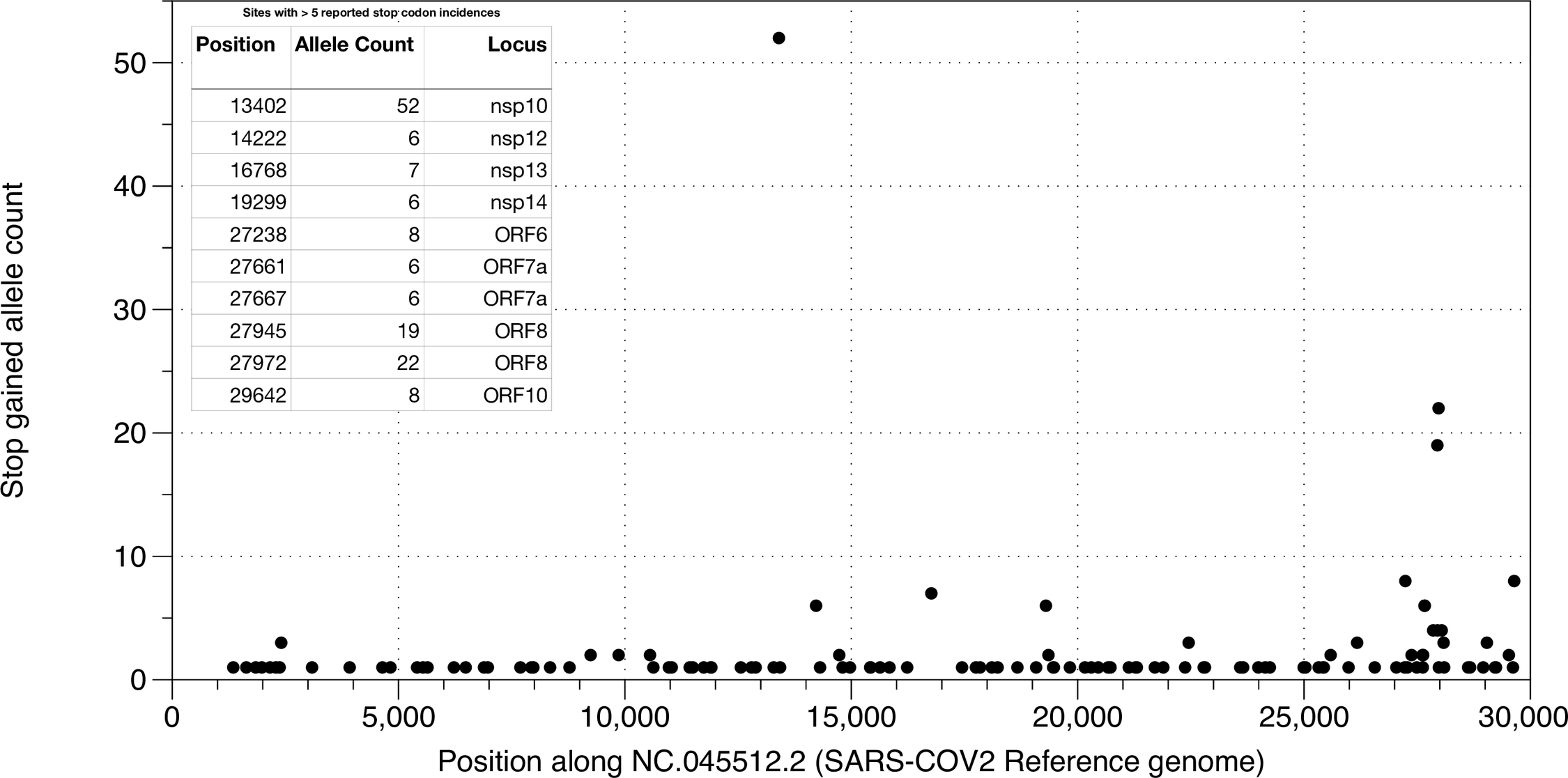

Supplement: S4 Fig — A snapshot of full length SARS-CoV-2 genome assemblies from GISAID and NCBI on 27 May 2020 was downloaded (comprising 39246 entries), and processed to detect single nucleotide variant alleles that introduced a stop codon. Introduced stop codons were detected in 270 entries, and the frequency of these alleles are plotted along the SARS-CoV-2 reference genome position. Introduced stop codons are rare but are distributed throughout the genomic sequences. Multiple loci harbor stop codons in unrelated assemblies. (TIF) [file pone.0244468.s004.tif]
